# Incidence of Daytime Sleepiness and Associated Factors in Two First Nations Communities in Saskatchewan, Canada

**DOI:** 10.3390/clockssleep1010003

**Published:** 2018-09-20

**Authors:** Chandima P. Karunanayake, James A. Dosman, Donna C. Rennie, Joshua A. Lawson, Shelley Kirychuk, Mark Fenton, Vivian R. Ramsden, Jeremy Seeseequasis, Sylvia Abonyi, Punam Pahwa, The First Nations Lung Health Project Research Team

**Affiliations:** 1 Canadian Centre for Health and Safety in Agriculture, University of Saskatchewan, 104 Clinic Place, Saskatoon, SK S7N 2Z4, Canada; 2 College of Nursing, University of Saskatchewan, 104 Clinic Place, Saskatoon, SK S7N 2Z4, Canada; 3 Department of Medicine, University of Saskatchewan, Royal University Hospital, 103 Hospital Drive, Saskatoon, SK S7N 0W8, Canada; 4 Department of Academic Family Medicine, University of Saskatchewan, West Winds Primary Health Centre, 3311 Fairlight Drive, Saskatoon, SK S7M 3Y5, Canada; 5 Community A, PO Box 96, Duck Lake, SK S0K1J0, Canada; 6 Department of Community Health & Epidemiology, College of Medicine, University of Saskatchewan, 107 Wiggins Road, Saskatoon, SK S7N 5E5, Canada

**Keywords:** subjective excessive daytime sleepiness, First Nations, house in need of repairs, dampness, money left over at the end of the month

## Abstract

Excessive daytime sleepiness (EDS) is the tendency to sleep at inappropriate times during the day. It can interfere with day-to-day activities and lead to several health issues. The objective of this study was to investigate the association between income, housing conditions, and incidence of EDS in adults living in two Cree First Nations communities. The data for this study involved 317 individuals aged 18 years and older who participated in baseline and follow-up evaluations (after four years) of the First Nations Lung Health Project, which was conducted in Saskatchewan in 2012–2013 and 2016. Both at baseline and follow-up survey after four years, an Epworth Sleepiness Scale (ESS) score >10 was considered to be abnormal. Logistic regression models were used to assess relationships between abnormal ESS and covariates at baseline. In 2016, 7.6% (24/317) of the participants reported an ESS >10 with the mean being 12.8 ± 2.0. For the same group, the mean ESS at baseline was 6.9 ± 2.2. The incidence of subjective EDS based on the ESS >10 was estimated at 7.6% over four years. This study showed an association between incidence of subjective EDS and less money left over at end of the month, having a house in need of repairs, having water or dampness in the past 12 months, and damage caused by dampness.

## 1. Introduction

Excessive daytime sleepiness (EDS) is the tendency to sleep at inappropriate times during the day. It can interfere with day-to-day activities such as work, school and/or relationships [1] and is associated with several health issues such as diabetes [2,3], heart disease [4,5], stroke [6], depression [7], and gastroesophageal reflux disease [8]. Motor vehicle accidents are the most dramatic consequence of EDS [9,10,11,12,13,14], with EDS accounting for 20% of total accidents. The prevalence of EDS in the general population varies from 9 to 28% [15]. Results from a pilot study reported subjective EDS prevalence to be 20.0% among rural Canadians [16]. Another study of rural Canadians reported the prevalence of subjective EDS was 15.9% in a population of adults (mean age 55 years) [17]. However, the prevalence of subjective EDS in two rural First Nations communities in Canada was reported to be 11% in a population of adults although younger than those in the rural population (mean age 35 years) [18].

Socio-economic factors impact daytime sleepiness [19,20]. Stringhini et al. [19] showed that women with a low position in their occupation (mainly unskilled workers) have a higher risk of subjective EDS. There was clear evidence of individuals with a lower socioeconomic gradient reporting greater daytime sleepiness [20].

Research has also shown that exposures to mold or dampness have been associated with adverse respiratory outcomes [21,22,23] and sleep problems [24,25,26] in both adults and children. In a study of adults living in England, participants living in damp buildings were more likely to report sleep disturbances [24]. In a study of Northern European adults, participants living in houses with reported signs of building dampness had a higher prevalence of insomnia and the association was strongest for floor dampness [25]. Two studies, one from Taiwan and one from Brazil, demonstrated an association between insomnia and EDS [27,28]. A recent study using the baseline data from this study [18] showed that a house in need of repairs, low annual household income and overcrowding were significantly associated with the prevalence of subjective EDS in First Nations communities. 

To our knowledge, the association between socio-economic status including income, housing conditions, and incidence of subjective EDS in First Nations adults has not been investigated. Thus, this study aimed to investigate the association between income, housing conditions and incidence of subjective EDS in adults living in two First Nations communities.

## 2. Results

Descriptive characteristics of the study population of 18 years and older (*n* = 317) can be found in Table 1. Of those who participated at baseline, the mean (±SD) age of men was 34.4 (±13.1) years and for women the mean age was 37.0 (±14.3) years. Twenty four percent of participants completed high school (Grade 12). Thirty one percent of participants were overweight and 36% were obese. Current smoking was present in 83.6%, ex-smoking in 6.9% and never smoking in 9.5% of participants. Loud snoring was reported by 16.1% of participants. In 2016, 7.6% (24/317) of participants reported an ESS > 10 with a mean subjective ESS of 12.8 ± 2.0 (Male: 13.2 ± 2.4; Female: 12.4 ± 1.6). For the same group, the mean subjective ESS at baseline was 6.9 ± 2.2 (Male: 6.8 ± 1.7; Female: 6.9 ± 2.6). The incidence of subjective EDS based on the ESS > 10 was estimated as 7.6% (Male: 7.9%; Female: 7.2%) over four years.

Certain baseline variables associated with the incidence of subjective EDS at *p* < 0.20 were considered as candidate variables in the multivariable model [29] and included chronic lung diseases, depression, money left over at the end of the month, having a house in need of repairs, water or dampness in the home in the past 12 months, damage caused by dampness, and mildew or moldy odor or musty smell. Table 2 presents the results from the multivariable analysis. The multivariable analysis was divided into three models because of the possible relationship between having a house in need of repairs and the indices of home dampness.

After adjusting for age, sex, BMI, and smoking status at baseline, not having enough money left over at end of the month and house in need of minor repairs were significant predictors for the incidence of subjective EDS (Table 2—Model 1). Also, after adjusting for age, sex, BMI, and smoking status at baseline, not having enough money left over at end of the month and water or dampness in the home in the past 12 months were significant predictors for the development of subjective EDS (Table 2—Model 2). After adjusting for age, sex, BMI, smoking status and money left over at end of the month at baseline, damage caused by dampness was significant predictor for the development of subjective EDS (Table 2—Model 3).

## 3. Discussion

The four-year incidence of subjective EDS in these two First Nations communities was found to be 7.6%. Not having enough money left over at end of the month, having a house in need of repairs, water or dampness in the home in the past 12 months, and damage caused by dampness were the main predictors of new cases of subjective EDS four years later.

Poor housing is a major concern in First Nations communities in Canada [30,31]. According to the 2016 Canada Census, one quarter (24.2%) of First Nations people lived in a dwelling that was in need of major repairs and 31.8% lived in dwellings that were in need of minor repairs. In 2016, 23.1% of First Nations people lived in crowded homes [30]. In this study, 41.4% of the participants lived in a dwelling that was in need of major repairs and 32.6% of the participants lived in crowded homes. In an earlier Canadian study with two other First Nations communities, First Nations’ houses were shown to be crowded with poor ventilation and the presence of mold [32].

According to the 2011 National Household Survey, the overall median income of First Nations participants was $23,600, and the median income for those with post-secondary qualifications was $33,100 [33]. Many First Nations people live well below the poverty line [34]. In the current study, 51.7% of the participants reported that they do not have enough money left over at the end of the month. Furthermore, those who did not have enough money left over at the end of the month or those who lived in a house in need of minor repairs also had a higher incidence of subjective EDS. Financial stress due to lack of work and poor health status can lead to insomnia [35,36]. Previous studies have shown financial stress and low income have been associated with subjective EDS [18,37].

The domestic environment of people living in poverty can lead to less than optimal sleep quality [38]. For example, poorly controlled temperature or high humidity can cause sleep deprivation [39]. People living in crowded homes where poverty also exists often need to share their meals and beds to sleep. Going to bed hungry or not having enough space for sleeping effects the quality of sleep, and the lack of sleep quality could lead to sleepiness during the day. 

In the current study, water or dampness in home during the past 12 months and damage caused by dampness were associated with the incidence of subjective EDS. In previous studies mold and dampness have been shown to be related to insomnia [24,25,26] and additionally, insomnia can lead to subjective EDS [40]. One possible mechanism that could explain the association between insomnia and building dampness is that building dampness can increase the emission of volatile organic compounds, impair air quality, dry mucous membranes, leading to sensory perceptions of worsening sleep quality [25]. Another mechanism that could explain this association is that dampness can cause nasal mucosal swelling and inflammation which could lead to impaired sleep quality [25]. The findings in this study were further supported by a study involving German children [26] in which exposure to visible mold or dampness in the home was observed increase the risk of insomnia related symptoms. It is possible that mold and home dampness could result in subjective EDS, both as a result of sleep deprivation due to insomnia, and/or due to irritation of the mucous membranes of the nose and oropharynx. 

### Limitations of the Study

This study had sample size of 317 and a less than optimal follow-up rate (45.2%). There were no differences in age and body mass index between follow-up and lost to follow-up participants. But, a significantly higher proportion of male participants were lost to follow-up. According to the Canadian Census of 2016 [41,42], the participants were not representative of the general population of these communities. There were more females and younger people who participated in the study and therefore, results cannot be generalized to populations of these communities. There were several major limitations of this study. Sleep status was measured subjectively by the Epworth Sleepiness Scale (ESS). There are differences between subjective (ESS) and objective sleepiness (e.g., multiple sleep latency test (MSLT)) measures. The ESS and MLST assess distinct aspects of sleepiness [43]. The ESS captured a subject’s self-reported level of sleep tendency in particular situation, on the other hand, MSLT used physiological data to assess the rate of falling asleep in an environment that is considered to be most suitable for sleep [43]. Another major limitation was that major sleep variables such as sleep duration, sleep disorders (as sleep-disordered breathing or insomnia), or the use of psychotropic drugs affecting sleep were not collected in this study. The measures of dampness and mold were self-reported and require objective confirmation. Lastly, although residents were living in the same community at both time points, there was a possibility that the participants were not living in the same home at the time of the second data collection. However, many of the homes of study participants were in need of major repairs. Thus, results should be interpreted with these limitations in mind.

## 4. Materials and Methods

### 4.1. Study Sample

**Figure 1:**
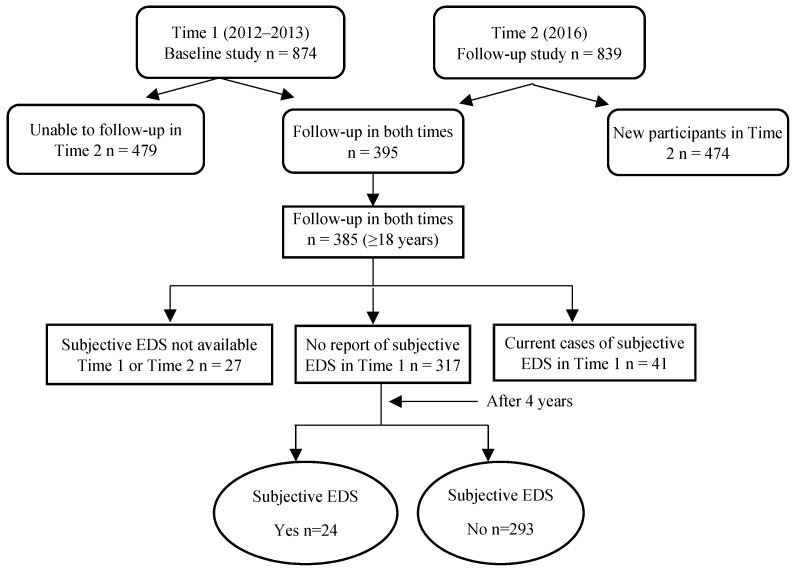
Study Sample.

The data for this study came from the baseline assessments and follow-up evaluations (after 4 years) as part of the First Nations Lung Health Project (FNLHP) conducted in two Cree First Nations communities (Community A and Community B) in Saskatchewan in 2012–2013 [44] and 2016. The original purpose of the FNLHP was to examine the predictors of respiratory health in First Nations populations and the investigation of sleep was a secondary consideration. There were 874 individuals who participated in the baseline survey and 839 who participated in a follow-up survey. The follow-up rate of those who participated at baseline and in the follow-up survey was 45.2% (395/874). A questionnaire was interviewer-administered to adult participants. Of those, 385 of them were aged 18 years and older. Data for subjective EDS were not available for 27 individuals at either baseline, follow-up, or both. There were 41 individuals who had been identified as current cases of subjective EDS at baseline. The prevalence of subjective EDS was 10.6% (41/385). After removing those cases (current cases and those lost to follow-up, *n* = 68), there were 317 individuals with no report of subjective EDS at baseline and who had participated in the follow-up questionnaire (Figure 1). The study was approved by the University of Saskatchewan’s Biomedical Research Ethics Board (Certificate No. Bio #12-89) and adhered to all of the criteria outlined in Chapter 9 entitled Research Involving the First Nations, Inuit, and Metis Peoples of Canada found in the Tri-Council Policy Statement: Ethical Conduct for Research Involving Humans [45]. Written consent was obtained from all participants.

### 4.2. Data Collection

Trained community research assistants conducted the baseline and the follow-up interviews at both time points. Adults 18 years and older were invited to the Community Health Centre to complete the interviewer-administered questionnaires and clinical assessments. This manuscript is based on the data from the questionnaire assessments. The Epworth Sleepiness Scale (ESS) [46,47,48,49] questionnaire was used to assess the degree of EDS. The ESS has not been validated in Australian Indigenous populations [50] or Canadian Indigenous populations. An ESS score >10 [17,46] was considered to be abnormal and was used to identify a case of subjective EDS at both at baseline and at the four-year follow-up. Independent variables of interest at baseline were self-reported age, sex, body mass index (BMI), education level, marital status, smoking status, alcohol consumption and employment status. “Doctor ever diagnosed” conditions included sinus trouble, heart problem, heart attack, tuberculosis, attack of bronchitis, emphysema, chronic bronchitis, COPD, asthma, diabetes, and depression. Other factors obtained through the questionnaire included respiratory symptoms such as chronic cough, chronic phlegm, shortness of breath (SOB), loud snoring, and money left over at end of the month. In addition, presence of environmental conditions in the home included state of house repairs; water or dampness from broken pipes, leaks, heavy rain or floods during the past 12 months; damage caused by dampness; mildew/moldy odor or musty smell; signs of mold or mildew; and number of persons per room as an index of crowding. For the analysis, the term “chronic lung diseases” was used to include one or more of emphysema, chronic bronchitis, chronic cough/chronic phlegm, and COPD.

### 4.3. Statistical Analysis

Statistical analyses were conducted using SPSS version 24 (IBM SPSS Statistics for Windows. Armonk, NY: IBM Corp., 2017). Logistic regression models were used to assess relationships between abnormal ESS and covariates at baseline. A multilevel logistic regression model using a generalized estimating equations approach was used to develop the model with individuals (first level) clustering within households (second level). The significant contributions of potential risk factors, confounders, and interactive effects were determined by developing a series of multilevel models. Variables with *p* < 0.20 in the univariate analysis became factors for the multivariable model [29]. In these analyses, the indicators of housing conditions were included separately. The variables retained in the final multivariable model included those that were statistically significant (i.e., *p* < 0.05) as well as age, sex, BMI, and smoking status. Odds ratios (ORs) and 95% confidence intervals (CIs) were used to present the strength of the associations.

## 5. Conclusions

The association between the incidence of subjective EDS and not having enough money left over at the end of the month, having a house in need of repairs, water or dampness in homes in past 12 months, and damage caused by dampness were novel findings and may be important public health issues. Further investigation into the mechanisms which influence these associations are necessary, while at the same time, provisions for addressing in-home dampness and mold are critical.

## Figures and Tables

**Table 1:** Univariable association between newly identified subjective EDS and demographic factors, co-morbid conditions, socio economic factors and housing environment.

Variables	Total *n* (%)	Newly Diagnosed Subjective EDS		*p* Value	Crude OR (95% CI)
		Yes *n* (%)	No *n* (%)		
**Demographics**					
Sex					
Male	151 (47.6)	12 (50.0)	139 (47.4)	0.812	1.11 (0.48, 2.55)
Female	166 (52.4)	12 (50.0)	154 (52.6)		1.00
Age group					
18–35 years	170 (53.6)	12 (50.0)	158 (53.9)	0.866	0.87 (0.18, 4.32)
36–55 years	122 (38.5)	10 (41.7)	112 (38.2)	0.976	1.02 (0.20, 5.15)
>55 years	25 (7.9)	2 (8.3)	23 (7.8)		1.00
Education level					
Less than high school	156 (49.4)	14 (58.4)	142 (48.6)	0.425	1.54 (0.53, 4.41)
Completed high school	77 (24.4)	5 (20.8)	72 (24.7)	0.907	1.08 (0.30, 3.93)
Post-secondary (university/technical or some)	83 (26.2)	5 (20.8)	78 (26.7)		1.00
Marital status					
Married/common law	119 (39.0)	8 (33.3)	111 (39.5)	0.550	0.77 (0.32, 1.83)
Widowed/divorced/separated/single	186 (61.0)	16 (66.7)	170 (60.5)		1.00
Body mass index					
Neither overweight nor obese	102 (31.1)	5 (20.8)	97 (34.2)	0.341	0.58 (0.19, 1.77)
Overweight	95 (30.9)	10 (41.7)	85 (29.9)	0.547	1.33 (0.52, 3.39)
Obese	111 (36.0)	9 (31.5)	102 (35.9)		1.00
Home crowding status					
More than one person per room	99 (32.6)	7 (31.8)	92 (32.6)	0.937	0.96 (0.39, 2.41)
One or less person per room	205 (67.4)	15 (68.2)	190 (67.4)		1.00
Alcohol consumption (5 or more drinks at a time)					
Never	61 (19.3)	4 (16.7)	57 (19.5)	0.675	0.78 (0.24, 2.51)
Occasionally	122 (38.6)	9 (37.5)	113 (38.7)	0.792	0.88 (0.35, 2.34)
Regularly	133 (42.1)	11 (45.8)	122 (41.8)		1.00
Smoking status					
Current smoking	265 (83.6)	20 (83.3)	245 (83.6)	0.638	0.74 (0.20, 2.64)
Ex-smoker	22 (6.9)	1 (4.2)	21 (7.2)	0.482	0.43 (0.04, 4.53)
Never smoker	30 (9.5)	3 (12.5)	27 (9.2)		1.00
Employment Status					
Employed (full-time/part-time/seasonally/self-employment)	73 (23.1)	3 (12.5)	70 (24.0)	0.182	0.42 (0.12, 1.49)
Student (part-time/full-time)	24 (7.6)	1 (4.2)	23 (7.9)	0.422	0.43 (0.05, 3.37)
Unemployed	219 (69.3)	20 (83.3)	199 (68.1)		1.00
**Co-morbid conditions**					
Shortness of breath					
Yes	156 (49.2)	13 (54.2)	143 (48.8)	0.607	1.24 (0.54, 2.83)
No	161 (50.8)	11 (45.8)	150 (51.2)		1.00
Loud snoring					
Yes	51 (16.1)	3 (12.5)	48 (16.4)	0.163	0.73 (0.21, 2.55)
No	265 (83.9)	21 (87.5)	244 (75.4)		1.00
Ever Dr. said					
Chronic lung diseases *					
Yes	81 (25.6)	9 (37.5)	72 (24.8)	0.163	1.84 (0.78, 4.35)
No	236 (74.4)	15 (62.2)	221 (75.2)		1.00
Sinus trouble					
Yes	85 (30.8)	7 (31.8)	78 (30.7)	0.908	1.05 (0.43, 2.58)
No	191 (69.2)	15 (68.2)	176 (69.3)		1.00
Heart problem					
Yes	25 (8.1)	1 (4.2)	24 (8.5)	0.456	0.47 (0.07, 3.39)
No	283 (91.9)	23 (95.8)	260 (91.5)		1.00
Tuberculosis					
Yes	24 (8.9)	1 (4.5)	23 (9.3)	0.469	0.47 (0.06, 3.65)
No	246 (91.1)	21 (95.5)	225 (90.7)		1.00
Attack of bronchitis					
Yes	81 (29.9)	5 (22.7)	76 (30.5)	0.444	0.67 (0.24, 1.86)
No	190 (70.1)	17 (77.3)	173 (69.5)		1.00
Asthma					
Yes	47 (14.8)	4 (16.7)	43 (14.7)	0.794	1.16 (0.38, 3.57)
No	270 (85.2)	20 (83.3)	250 (85.3)		1.00
Diabetes					
Yes	42 (13.8)	5 (20.8)	37 (13.2)	0.310	1.74 (0.60, 5.04)
No	263 (86.2)	19 (79.2)	244 (86.8)		1.00
Depression					
Yes	61 (19.9)	7 (29.2)	54 (19.1)	0.238	1.75 (0.69, 4.42)
No	246 (80.1)	17 (70.8)	229 (80.9)		1.00
Ear infection					
Yes	44 (14.1)	4 (16.7)	40 (13.9)	0.716	1.24 (0.39, 3.88)
No	268 (85.9)	20 (83.3)	248 (86.1)		1.00
Heart burn/Stomach Reflex					
Yes	50 (16.0)	5 (21.7)	45 (15.5)	0.440	1.51 (0.53, 4.31)
No	263 (84.0)	18 (78.3)	253 (84.5)		1.00
**Socio-economic factors**					
Money left over at the end of month					
Not enough money	156 (51.7)	17 (77.3)	139 (49.6)	0.072	3.09 (0.91, 10.57)
Just enough money	67 (22.2)	2 (9.1)	65 (23.2)	0.784	0.78 (0.13, 4.60)
Some money	79 (26.1)	3 (13.6)	76 (27.1)		1.00
**Housing conditions**					
House in need of repairs					
Yes (major repair)	123 (41.4)	10 (45.5)	113 (41.1)	0.086	3.86 (0.83, 18.04)
Yes (minor repair)	85 (28.6)	10 (45.5)	75 (27.3)	0.026	5.82 (1.23, 27.52)
No (only regular maintenance)	89 (30.0)	2 (9.1)	87 (31.6)		1.00
In past 12 months, water or dampness in home					
Yes	182 (63.4)	18 (85.7)	164 (61.7)	0.035	3.73 (1.10, 12.66)
No	106 (36.6)	3 (14.3)	102 (38.3)		1.00
Damage caused by dampness					
Yes	153 (48.3)	16 (66.7)	137 (46.8)	0.061	2.28 (0.96, 5.39)
No	164 (51.7)	8 (33.3)	156 (53.2)		1.00
Mildew/moldy odor or musty smell					
Yes	153 (53.9)	16 (72.7)	137 (52.3)	0.075	2.43 (0.91, 6.46)
No	131 (46.1)	6 (27.3)	125 (47.7)		1.00
Signs of mold or mildew in home					
Yes	133 (48.0)	12 (57.1)	121 (47.3)	0.385	1.49 (0.61, 3.66)
No	144 (52.0)	9 (42.9)	135 (52.7)		1.00

* We used the term “chronic lung diseases” to include one or more of emphysema, chronic bronchitis, chronic cough/chronic phlegm and COPD.

**Table 2:** Multivariable association between newly identified subjective daytime sleepiness and risk factors.

	Model 1 ^†^		Model 2 ^†^		Model 3 ^†^	
	Adjusted OR (95% CI)	*p* Value	Adjusted OR (95% CI)	*p* Value	Adjusted OR (95% CI)	*p* Value
**Socio-economic factors**						
Money left over at the end of month						
Not enough money	3.83 (1.02, 14.43)	0.047	4.62 (1.11, 19.32)	0.036	3.32 (0.93, 11.79)	0.064
Just enough money	0.85 (0.13, 5.63)	0.873	1.08 (0.16, 7.43)	0.937	0.69 (0.12, 4.12)	0.684
Some money	1.00		1.00			
**Housing conditions**						
House in need of repairs						
Yes (major repair)	4.47 (0.86, 23.14)	0.074				
Yes (minor repair)	5.72 (1.10, 29.73)	0.038				
No (only regular maintenance)	1.00					
In past 12 months, water or dampness in home						
Yes			3.54 (1.02, 12.22)	0.046		
No			1.00			
Damage caused by dampness						
Yes					2.79 (1.02, 7.65)	0.046
No					1.00	

† Results are presented as adjusted odds ratios (OR) and 95% confidence intervals (95% CI). Adjusted for age, sex, body mass index, smoking status, and variables in the table. Correlation between dampness and repair is 0.356 (Spearman’s rho), and correlation between damage caused by dampness and repair is 0.444 and moderately correlated. Correlation between dampness and damage caused by dampness is 0.612 and highly correlated.

## Abbreviations

EDS	Excessive daytime sleepiness
ESS	Epworth Sleepiness Scale
FNLHP	First Nations Lung Health Project
BMI	Body Mass Index
COPD	Chronic Obstructive Pulmonary Disease
SOB	Shortness of Breath
SD	Standard Deviation
MSLT	Multiple Sleep Latency Test
